# Quality of care in children with chronic diseases

**DOI:** 10.1186/1824-7288-40-S1-A36

**Published:** 2014-08-11

**Authors:** Paola Rucci, Elisa Zanello, Davide Roccaro, Simona Calugi, Giulia Pieri, Maria Pia Fantini

**Affiliations:** 1 Department of Biomedical and Neuromotor Sciences, University of Bologna, Bologna, Italy

## Background

Improving health care for chronic health conditions is a major goal of contemporary health service delivery systems. To date, the main research focus has been on adults and elderly, while fewer attention has been focused on newborns and children with chronic health conditions. To address the challenges related to the provision of integrated care to children with special health care needs, the Special Needs Kids (SpeNK) project was carried out in Emilia-Romagna Region. The specific aims of this study were: to review the ongoing sheltered discharge procedures, to develop and test instruments to assess the families’ perspective on the continuity of care and to estimate the time devoted by the family pediatrician to care coordination activities.

## Materials and methods

The SpeNK project is still ongoing. After reviewing the procedures implemented in the Local Health Authorities of the study area, 10 face-to-face, 3 telephone semi-structured interviews and a focus group with the families at 1-6 months from discharge were conducted by a psychologist. A 20-item questionnaire on continuity of care was then developed, based on the contents of the interview and on Haggerty’s constructs of informational, management and relational continuity. The questionnaire was validated on 102 parents of preterm newborns and then administered by phone to the families of children enrolled in the SpeNK study after 9 months from discharge.

## Results

A qualitative analysis of the contents of the semi-structured interviews revealed that families underscored the importance of informational continuity among hospital clinicians and across health care services, set a high value on the information/training received during the hospital stay of children and at discharge, and exhibited a mixed attitude towards involvement in the health care decisions.

Examination of the structure of the questionnaire using factor analysis with oblique rotation identified 5 factors accounting for 61.1% of the variance of items. The factors identified can be interpretable as ‘management continuity’ , ‘informational continuity’ ‘trustful relation with the family pediatrician’, ‘information provided to families’ and ‘family empowerment’.

## Conclusions

The preliminary results of the qualitative analysis of the semi-structured interviews suggest the relevance to the families of the hospital experience and some criticalities in the informational continuity among professionals. The questionnaire on continuity of care has a well-defined structure and can be a useful tool to capture problems related to informational continuity and to the interaction of families with the professionals involved in the care of children with special care needs.

